# Is Intravitreal Injection of Recombinant Tissue Plasminogen Activator Effective for the Treatment of Refractory Diabetic Macular Edema in Patients With Posterior Vitreous Detachment?

**DOI:** 10.7759/cureus.54297

**Published:** 2024-02-16

**Authors:** Şule Berk Ergun, Yasin Toklu, Mücella Arıkan Yorgun, Melek Mutlu Sönmez, Hasan B Arifoğlu, Hasan B Çakmak

**Affiliations:** 1 Department of Ophthalmology, Bilkent City Hospital, Ankara, TUR; 2 Department of Ophthalmology, Yıldırım Beyazıt University Faculty of Medicine, Ankara, TUR; 3 Department of Ophthalmology, Göztepe City Hospital, İstanbul, TUR; 4 Department of Ophthalmology, Okan University Faculty of Medicine, İstanbul, TUR; 5 Department of Ophthalmology, 100.Yıl Hospital, Ankara, TUR

**Keywords:** best-corrected visual acuity, central macular thickness, recombinant tissue plasminogen activator, posterior vitreous detachment, refractory diabetic macular edema

## Abstract

Objective: To determine whether intravitreal injection of recombinant tissue plasminogen activator (rTPA) is effective for the treatment of refractory diabetic macular edema (DME) in patients who already had posterior vitreous detachment (PVD).

Methods: It is a retrospective chart review of the patients with refractory DME and PVD. The efficacy of intravitreal injection of rTPA was assessed based on the changes in central macular thickness (CMT) and best-corrected visual acuity (BCVA) in these patients.

Results: Nine eyes of nine patients as the study group and 14 eyes of the 14 patients as the control group were examined. Before the injections, the mean CMT was 470.0± 107.6 in the study group, compared to 536.2± 150.5 in the control group, with no statistical significance (p=0.403). The statistical analysis revealed no significant differences in the mean changes in CMT from baseline to one and three months after injections between the study and control groups (p=0.439, p=0.781, respectively). Likewise, no statistically significant disparities were observed in the mean pre-injection BCVA between the study group (0.877± 0.349) and the control group (0.950± 0.300) (p=0.415). Additionally, after three months of injection, there were no significant changes in the mean BCVA of the study group (0.844± 0.304) and the control group (0.864± 0.253) (p=0.512).

Conclusion: This study showed that rTPA has no effect on changes in CMT and BCVA in patients who had refractory DME and PVD at the same time. This may suggest that the improvement in CMT in previous studies may be due to the induction of PVD.

## Introduction

Refractory diabetic macular edema (DME) is a big concern and is the most common cause of visual impairment in diabetic patients [1]. Various treatment approaches have been utilized to address DME, such as focal/grid-pattern photocoagulation, the use of intravitreal corticosteroids, and anti-vascular endothelial growth factor agents [2]. Additionally, pars plana vitrectomy and detachment of the posterior hyaloid may prove beneficial in cases with evident posterior hyaloid traction [3,4]. However, there is a rising curiosity surrounding alternative treatment methods for refractory DME. Among these, pharmacologic vitreolysis agents have gained attention [5,6].

The incidence of DME in eyes with posterior vitreous detachment (PVD) is lower when compared to those with a thickened and adhered hyaloid membrane where the macular traction of the vitreous cortex is likely to play a significant role in the pathogenesis of the macular edema [7,8]. It was also demonstrated that traction forces significantly contribute to the pathogenesis of DME and diminish the efficacy of intravitreal anti-VEGF therapy in eyes with vitreoretinal interface abnormalities [9].

Could intravitreal plasmin or recombinant tissue plasminogen activator (rTPA) contribute to edema resolution solely through inducing PVD, or does it exert additional effects by releasing remaining attachments subsequent to PVD?

This study aims to evaluate the effectiveness of intravitreal rTPA injections in treating refractory DME among patients who have previously undergone PVD, assessing its efficacy through alterations in central macular thickness (CMT) and best-corrected visual acuity (BCVA).

## Materials and methods

This is a proof-of-concept study utilizing a retrospective chart review approach to analyze the medical records of patients diagnosed with refractory DME and PVD who attended the clinic over a one-year period. The study group encompassed patients who had undergone intravitreal injections of 25 micrograms of rTPA, while the control group comprised individuals who had declined intravitreal rTPA treatment and opted for follow-up; selected through convenience sampling. Written informed consent for injection procedure was obtained from these patients before the injections and the study adhered to the tenets of the Declaration of Helsinki. The study was approved by our institutional ethics committee (E1-22-2928). The main outcome measures were CMT determined by optical coherence tomography (OCT) and BCVA.

Participants qualified for the study if they met the following criteria: Type-2 diabetes mellitus with nonproliferative diabetic retinopathy, refractory DME, and confirmed PVD diagnosed through biomicroscopy using a +90 D lens and/or OCT. Exclusion criteria encompassed a history of intraocular surgery within the previous six months, intravitreal injections and/or laser treatments within the past four months, and a medical history of cardiovascular or cerebrovascular events.

Refractory DME was defined as resistant edema through previous lines of treatment including laser, three consecutive doses of anti-vascular endothelial growth factor agents, and at least one intravitreal triamcinolone acetonide injection.

Commercially available rTPA (Actilyse, Boehringer-Ingelheim, Biberach, Germany) was diluted to solutions of 25 micrograms/0.1 ml, divided into multiple aliquots of 0.2 ml in insulin syringes and kept frozen at -80 °C. Using a sterile technique, the solution was prepared by injecting 4 ml of the provided water for injection diluent into the 10 mg Actilyse vial. Subsequently, 1 ml of this solution was drawn up, and it was augmented to a total volume of 10 ml using 0.9% sodium chloride for injection. The solution was made to reach room temperature around about 30 minutes before the injections. Each syringe was used for one patient after the change of the needle and the adjustment of the dosage. After topical anesthesia, 0.1 ml of the solution was injected through pars plana, inferotemporal quadrant, 3.5 or 4 mm away from the limbus depending on whether the patient was pseudophakic or phakic, respectively, under sterile conditions in the operating room. After the injections, topical antibiotics were prescribed four times a day for one week.

For each patient routine ocular examinations including BCVA, applanation tonometry, biomicroscopy, fundus examination by indirect ophthalmoscope, and OCT were performed prior to, one month after, and three months after rTPA injections. To minimize the effect of diurnal variation, the scans were performed at the same time of the day (between 9.00 and 11.00 am). BCVA was recorded by using a Snellen chart and then converted to the logarithm of the minimum angle of resolution (LogMAR) for statistical purposes.

Data were statistically analyzed with the Statistical Package for Social Sciences (SPSS), version 22.0 (IBM Corp., Armonk, NY). The normality assumption was checked by the Shapiro-Wilk test and it was found that the data did not show normal distribution. Comparisons between groups were performed by the Mann-Whitney U test. For comparing categorical data, a chi-square test was performed. P<0.05 was considered to be statistically significant.

## Results

In total, 23 patients with refractory DME and PVD were enrolled in this study. Nine eyes of nine patients (three female, six male) as the study group, and 14 eyes of the 14 patients (six female, eight male) as the control group were examined (p=0.543). The mean age of the patients was 64.3±7.8 in the study group and 61.4±7.1 in the control group. No significant difference in age was present between the groups (p=0.677) (Table 1). No ocular or systemic complications were seen related to injections. 

**Table 1:** Baseline demographics and clinical characteristics of the patients. BCVA: best-corrected visual acuity, CMT: central macular thickness, logMAR: logarithm of the minimum angle of resolution, rTPA: recombinant tissue plasminogen activator.

	rTPA group (n: 9)	Control group (n: 14)	p value
Age, years	64.3 ± 7.8	61.4 ± 7.1	0.677
Female, n (%)	3 (33 %)	6 (42 %)	0.542
Baseline BCVA, logMAR	0.8 ± 0.3	0.9 ± 0.3	0.415
Baseline CMT, μm	470.0 ± 107.6	536.2 ± 150.5	0.403

Before the injections, the mean CMT was 470.0± 107.6 μm in the study group, compared to 536.2 ± 150.5 μm in the control group. The mean CMT of the control group was slightly greater than that of the study group, although statistically not significant (p=0.403) (Table 1).

One month after the injections, the CMT was 457.4 ± 137.1 μm in the study group and 586.8 ± 178.6 in the control group. Three months after the injections, the CMT was 473.3 ± 125.3 μm in the study group and 502.5 ± 143.8 μm in the control group. There were no statistically significant differences between the two groups in the first-month and third-month examinations of the mean values of CMT, respectively (p=0.109, p=0.926) (Table 2).

**Table 2:** Mean CMT and BCVA values throughout the follow-up compared with the study groups. BCVA: best-corrected visual acuity, CMT: central macular thickness, logMAR: logarithm of the minimum angle of resolution, rTPA: recombinant tissue plasminogen activator.

		rTPA group (n: 9)	Control group (n: 14)	p value
BCVA, logMAR			
	Baseline	0.8 ± 0.3	0.9± 0.3	0.415
	1^st^ month	0.7 ±0.3	0.8 ±0.3	0.716
	3^rd^ month	0.8 ±0.3	0.8± 0.2	0.512
CMT, μm				
	Baseline	470.0± 107.6	536.2 ± 150.5	0.403
	1^st^ month	457.4 ± 137.1	586.8 ± 178.6	0.109
	3^rd^ month	473.3 ± 125.3	502.5 ± 143.8	0.926

No statistically significant differences were found between the mean preinjection BCVA of the study group (0.8 ± 0.3 logMAR) and the control group (0.9± 0.3 logMAR) (p=0.415) (Table 2). Three months after injections, no significant changes were observed in the mean BCVA of the study group (0.8 ±0.3 logMAR) and the control group (0.8± 0.2 logMAR) (p=0.512) (Table 2).

The mean changes in lines of CMT and BCVA from baseline to three months after injections were also not statistically different between the study group and the control group (p=0.439, p=0.781 respectively) (Table 3).

**Table 3:** The changes in lines of mean CMT and BCVA values from baseline to 1st and 3rd months compared with the study groups. BCVA: best-corrected visual acuity, CMT: central macular thickness, logMAR: logarithm of the minimum angle of resolution, rTPA: recombinant tissue plasminogen activator.

		rTPA group (n: 9)	Control group (n: 14)	p value
Change in BCVA, logMAR				
	1^st^ month	-0.09 ± 0.06	-0.08 ± 0.05	0.924
	3^rd^ month	-0.03 ± 0.06	-0.08 ± 0.05	0.472
Change in CMT, μm	1^st^ month	-12.66 ± 44.96	50.57 ± 34.93	0.439
	3^rd^ month	8.88 ± 20.05	-33.71 ± 31.73	0.781

## Discussion

Many macular diseases involve tractions at the vitreoretinal interface, often mediated by fibrocellular proliferation in this area [10]. Several studies have highlighted the significance of vitreoretinal interface changes in the formation of macular holes, epiretinal membranes, and vitreomacular traction. Additionally, it has been demonstrated that traction forces play a significant role in the pathogenesis of DME and reduce the effectiveness of intravitreal anti-VEGF therapy in eyes with vitreoretinal interface abnormalities [9]. Therefore, the complete removal of the cortical hyaloid by mechanical or pharmacological means is usually the principal goal for vitreoretinal surgeons [11,12]. However, cortical vitreous fibrils of varying extend persist even after detachment of the hyaloid [13,14]. This incomplete vitreoretinal separation is usually described in diabetic eyes more than in nondiabetic eyes because of the effects of diabetes on the macromolecules of vitreous and the structural consequences [15,16].

rTPA, a genetically engineered serine protease, catalyzes the conversion of plasminogen into plasmin, a critical enzyme in fibrinolysis that breaks down various extracellular matrix components like fibronectin and laminin, crucial at the vitreoretinal interface and implicated in retinal adhesions [17,18]. There are also some studies reporting the use of tissue plasminogen activator (TPA) for macular edema associated with central retinal vein occlusion [19,20], refractory DME [21], and plasmin for DME [6,22-24].

Hesse et al. first reported that the injection of intravitreal TPA induces PVD [25,26]. Murakami et al., in their study, speculated that PVD induced by intravitreal TPA injection might be a possible mechanism to resolve macular edema in central retinal vein occlusion [19].

Though chemical vitreolysis is commonly employed to induce PVD for various purposes, limited studies exist regarding its specific impact on refractory DME, notably the study by Diaz-Llopis et al., utilizing plasmin for chemical vitreolysis and demonstrating reduced CMT across all patients [6]. The study by Abrishami et al., the first to evaluate the effects of induction of PVD by intravitreal TPA injection for the refractory DME, successfully demonstrated PVD induction in patients [21]. It is important to note that both studies excluded cases with previous PVD.

Based on these findings, intravitreal rTPA injection might improve VA outcomes and induce resolution of macular edema by means of inducing PVD. But what, if the patient already has PVD, is there still a positive effect of releasing remaining tractions at the vitreoretinal interface? This action could potentially contribute to further improvement in the patient's condition, albeit the exact extent of this effect warrants further investigation.

Indeed, Kishi et al. highlighted the persistence of vitreous cortex remnants even after spontaneous PVD [27]. The suggestion of differences between TPA-induced and spontaneous PVD by Murakami et al. aligns with the discovery that enzymatically induced PVD effectively eliminates these remnants by Asami et al. [5,19]. Eliminating those remaining traction forces affecting the retina may be helpful in patients who already have posterior vitreous detachment.

This fact made us think about whether the intravitreal rTPA might have additional effects on the resolution of edema along with PVD. However, the study outcomes revealed that intravitreal injections of rTPA had no effect on changes in CMT and BCVA in patients with refractory DME who had already had PVD at the same time. This may support the findings in previous studies, which suggest that the improvement in CMT may be due to the induction of PVD. However, there exists a case report demonstrating substantial CMT improvement following intravitreal TPA for treatment-resistant DME in a vitrectomized eye [28], suggesting a potential variance in responses among specific cases or conditions.

The most significant limitation of this study lies in its small sample size and retrospective design. However, it's important to note that this study stands as the first study within the literature utilizing rTPA in such a specific and isolated group, despite these limitations. As it is known; PVD has been shown in 63% of patients without macular edema and in 31% of those with macular edema [29]. Hikichi et al. also found spontaneous improvement in macular edema as a result of separation of the vitreous from the macula [7]. Hence, encountering cases of refractory DME in individuals who have already experienced PVD is quite challenging. The limited number of cases in this study makes it challenging to generalize results; however, these findings could serve as a guiding light for future studies involving larger cohorts to shed more comprehensive insights.

Managing cases presenting with both PVD and refractory DME remains controversial within the field. Another critical aspect yet to be addressed is the outcomes following repeated injections. While this study wasn't specifically designed to address this aspect, future studies dedicated to investigating this issue, particularly focusing on the safety and appropriate dosages of rTPA, could provide valuable insights and clarification on the matter.

## Conclusions

Encountering cases of refractory DME in individuals who have already experienced PVD is quite challenging, resulting in a restricted patient pool for the study. Despite this limitation, few data in the literature on this subject increases the importance of the study.The study results indicating that rTPA had no impact on CMT and BCVA in such patients might imply that previous improvements in CMT could be attributed to induced PVD. However, further investigations are essential to confirm and validate these findings, emphasizing the necessity for additional studies in this domain.
